# Supplementary material for: Crevice Corrosion Behavior of 201 Stainless Steel in NaCl Solutions with Different pH Values by In Situ Monitoring
Source: Materials (Basel). 2024 Mar 1;17(5):1158. doi: 10.3390/ma17051158 (PMC10933831; doi:10.3390/ma17051158)
Supplement: Supplementary file 1 [file materials-17-01158-s001.zip › materials-2845139-supplementary.pdf]

Supporting Information

Crevice Corrosion Behavior of 201 Stainless Steel in NaCl Solutions with Different pH Values by In Situ Monitoring

Zejie Zhu [1,*], Hang Zhang [1], Yihan Bai [1], Pan Liu [2,*], Haoran Yuan [1,*], Jiangying Wang [1] and Fahe Cao [3]

[1] School of Materials and Chemistry, China Jiliang University, Hangzhou 310018, China; zhanghcjlu@163.com (H.Z.); baiyhcjlu@163.com (Y.B.); wangjiangying@cjlu.edu.cn (J.W.)
[2] Frontier Research Initiative, New Industry Creation Hatchery Center (NICHe), Tohoku University, Sendai 980-8579, Japan
[3] School of Materials, Sun Yat-Sen University, Shenzhen 518107, China; caofh5@mail.sysu.edu.cn
* Correspondence: zejiezhu@cjlu.edu.cn (Z.Z.); liu.pan.a5@tohoku.ac.jp (P.L.); mcdhyhr@cjlu.edu.cn (H.Y.)

Table of Contents

1. **Electrochemical measurements** **S-2**
2. **SECM test** **S-3**

1. Electrochemical measurements

All electrochemical measurements were carried out at using an electrochemical workstation (CHI920D, Shanghai, China) at 25 ºC. The open circuit potential (OCP) and polarization measurements were carried out using a 201-SS as the working electrode (WE1), a Pt wire as the counter electrode and a homemade reference electrode (RE1): an Ag/AgCl electrode was put into a test solution-filled PTFE tube (containing a porous ceramics cap at the end). All the potentials recorded in the test were transformed into potentials against Ag/AgCl electrode (0.206 V vs. standard hydrogen electrode at 24.85℃). The OCP was tested for 900 s, after 0.5, 1, 2, 4, 6, 8, 24 and 48 h immersion. The polarization started after the OCP was stabilized (about 30 min, ΔOCP < 10 mV) with a scan rate of 1 mV/s by positively scanning the potential from -0.2 V_{OCP} to +0.35 V_{OCP}.

2. SECM experiment

A commercial SECM system, the CHI 920D (CH Instruments, Inc., Shanghai, China), was used to perform the SECM experiments. The SECM system consisted of a stepper motor and a bipotentiostat. The 201-SS samples were left at their respective open circuit potentials throughout the experiments. The SECM experiment was performed with a three-electrode configuration using an Ag/AgCl/KCl (3.0 M) as the reference electrode, a Pt wire as the counter electrode, and a submicron Pt/IrO_x-pH UME as the working electrode. The diameters of the three holes for pH measuring were designed to be 100 μm, the distance between two holes was 2.7 mm in the current work. The pH UME was first calibrated before the experiment by observing the response of the potential in a variety of buffer solutions. Before the SECM experiment, the pH UME was immersed in a buffer solution with a pH of 7 for 24 hours. In this way, a stable hydrated film would form at the tip of the pH UME. The distance between the tip and the substrate was controlled by gently resting the probe on the sample as seen through an optical microscope (A0-V128S, AVSVI, Shenzhen, China). The Z-positioning motor was operated at a slow speed (0.3 μm/s) to reduce the vibration of the SECM stage. The radial pH distribution within the gap was measured using a potentiometric mode of the SECM. **Figure S1** shows the arrangement of the SECM cell for pH measurement. It should be noted that the tip for pH measurement was first positioned at the hole near the opening. Then, the tip was lifted and moved to the hole in the middle and at the bottom using the positioning motor to measure pH. The distance between the tip and substrate was 10 μm when measuring the pH values inside crevice.

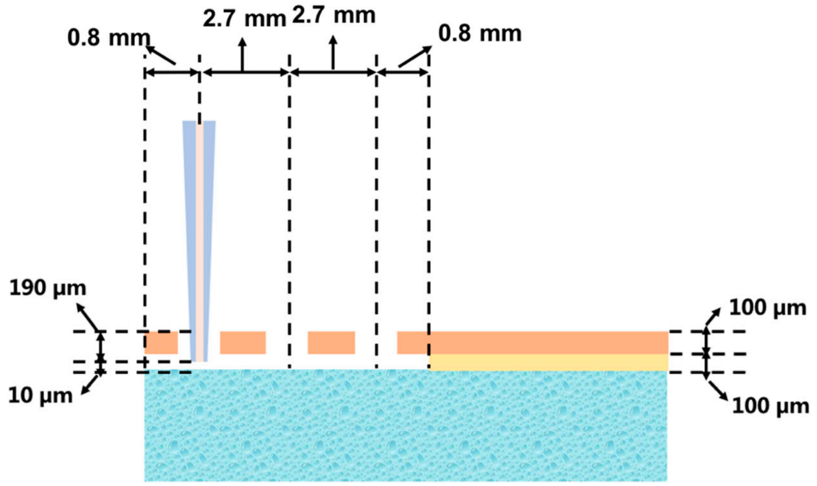

Figure S1 The SECM cell arrangement for pH measurements.
